# Case Illustrative of the Beneficial Effects of the Carbonate of Iron in Tic Douloureux

**Published:** 1821-09

**Authors:** 

**Affiliations:** Member of the Royal College of Surgeons.


*Case illustrative of the beneficial Effects of the Carbonate of Iron in Tic Douloureux.* By Mr. Richmond, Member of the Royal College of Surgeons.

MRS. VICARS, of Marsh Chappel, a lady about thirty years of age, of delicate fibre and thin habit, applied to me about a year ago, with a severe and very acute pain along that branch of the fifth pair of nerves passing to the maxilla inferior, and particularly assailing the mental nerve. This pain she describes as coming on at intervals, with such a degree of severity as to amount to little less than distraction, and to produce very great mental inquietude and apprehension. Upon a more minute investigation of the case, it appeared that, about eleven years ago, she received a stroke from a cow on the same side of the lower jaw to which she referred the pain, and that an exfoliation took place soon afterwards. From this period she was quite free from pain, until about four years ago, when her affliction came on, and all the symptoms denoted the most marked and tormenting case of tic douloureux.

Being fully aware of the general inefficacy of dividing the nerve, I neither attempted nor proposed it; but, in conformity with the advice of Mr. Abernethy, I endeavoured to soothe constitutional irritation by promoting healthy secretion from the organs connected with digestion. Leeches and blisters were also applied; a treatment which evidently produced a certain degree of alleviation for a short period. Having perused Mr. Benjamin Hutchinson's pamphlet, and witnessed the beneficial effects of his practice in tic douloureux, I again visited the patient, and found her agonies in all their former violence. She was advised to try the carbonate of iron, one drachm of which she took three times a-day. On having taken two dozen of the powders, she experienced very great amendment, but exclaimed that I had been salivating her: indeed the iron, according to her account, had produced considerable ptyalism;* but, from its having subsided within a few days after she ceased to take the powders, during which period I did not see her, I am unable to form any judgment of its extent. I urged her not to regard the trifling inconvenience of a sore mouth, but to persevere in the use of the medicine; which she assured me she would most gladly do, as her excruciating torments had been so very considerably relieved. She further expressed great comfort in the improvement of her tongue and mouth, which were foul and unpleasant before she took the iron; and, though it occasioned some sense of weight and uneasiness in the stomach, her appetite and bodily strength had very much increased. She has regularly continued the medicine until last week, when the pain had totally ceased, and has not recurred, to the present time.

Were I to attempt to account for the *modus operandi* of the carbonate of iron in this disease, I should refer it to a theory which, in the full extent in which it has been proposed, many admire and many condemn,—I allude to the production of local disease from disordered digestion, according to the principles of Mr. Abernethy. I am induced to suppose, from the improvement of the tongue, of digestion, and of mental energy, that the iron acts not only immediately upon the blood by entering into its composition, but also consecutively, by promot-

---

* This effect of iron is unusual, but will sometimes occur: I believe that Mr. Hutchinson has witnessed a similar occurrence two or three times.

ing the energy and healthy action of the stomach, and the organs therewith connected, which we know must produce commensurate effects upon the sensorium and its connexions.

*Grimsby, Lincolnshire; June,* 1821.

